# Burnout in primary healthcare providers: A cross-sectional study from Al-Khobar, Saudi Arabia

**DOI:** 10.1371/journal.pone.0352977

**Published:** 2026-07-10

**Authors:** Banan Almalki, Hani Alshehri, Lateefah Alharbi, Sarah Aldossary, Amira Alshowkan

**Affiliations:** 1 Senior Registrar, Eastern Health Cluster, Dammam, Saudi Arabia; 2 Family Medicine and mental health specialist, Eastern Health Cluster, Dammam, Saudi Arabia; 3 Family Medicine Consultant, Eastern Health Cluster, Dammam, Saudi Arabia; 4 Assistant Professor, College of Nursing, Imam Abdulrahman Bin Faisal University, Dammam, Saudi Arabia; West University of Timisoara: Universitatea de Vest din Timisoara, ROMANIA

## Abstract

**Aim:**

Job burnout is one of the emerging challenges in the healthcare sector which caused various issues among the primary healthcare providers. Hence, the present study was designed to evaluate the prevalence of burnout among the healthcare workers working in the primary healthcare clinics situated in the Alkhobar, Eastern Region of Saudi Arabia.

**Methods:**

The study was also aimed to evaluate the associated factors related to the burnout. This cross-sectional study included 114 healthcare providers working in the different primary health care centers. Maslach Burnout Inventory (MBI) was used to evaluate the burnout among the respondents.

**Results:**

The other questions were included the demographics and clinical factors. It was found that the prevalence of the burnout was 21.1%. Emotional exhaustion was found significantly high among females compared to males and those who had monthly income less than 12K compared to those with more than 12K monthly income. Daily patient load was another factor causing emotional exhaustion among the respondents.

**Conclusion:**

In conclusion, the feeling of depersonalization among the participants was found to be very high followed by emotional exhaustion. The identification of the contributing factors would help to reduce the prevalence. Some of those factors were socio-demographic related and some were related to work.

## Introduction

The role of the primary health care centers (PHCCs) is to provide services to treat or prevent diseases and rehabilitation. This role helps in reducing the disease burden and improve the health of the population [1,2]. However, to provide best healthcare services at the PHCCs, it is vital to have competent and motivational doctors and staff to provide high-quality services. On the other hand, the structure of the healthcare system which leads to heavy patients load on the PHCCs may lead to burnout [3].

Job burnout is a considerable challenge among the health workers [4]. It has potential negative consequences on the mental health of health care providers, the patients as well as the organization [5–7]. Studies reported that poorer work performance, lower job commitment and higher turnover intention are caused due to the job burnout [8,9]. Therefore, to evaluate the prevalence of job burnout among the healthcare workers would help to improve their mental health and reduce the burnout [10].

Maslach in 1980s defined that the occupational burnout is a psychological state and it is an end state of chronic stress associated with work [11]. Therefore, Maslach developed an inventory named Maslach Burnout Inventory (MBI) which consisted of three sections [12]. Emotional exhaustion (EE) refers to depletion of emotional energy because of the continued work demands. Depersonalization (DP), it occurs due the EE and it makes emotionally detached from the work or job. The third section of MBI is low personal accomplishment (PA), it refers that the sense of self-worth or efficacy related to work is reduced among the healthcare workers.

Numerous studies have been conducted to evaluate the prevalence of job burnout among the healthcare professionals. In the United States, the prevalence of burnout among the medical students was reported between 31% and 49.6%, 30% among physicians and 50% to 76% among residents [13,14]. In the study published from Saudi Arabia reported the 32% prevalence of burnout among the family medicine residents [15]. However, prevalence of burnout was found to be 12.6% among the family medicine physicians working in Qatar [16] and it was as high as 43% in the European countries [17].

To the authors’ best knowledge, very limited studies have been published from the Eastern Region of Saudi Arabia. Recent studies in Saudi Arabia have reported varying burnout rates among primary care physicians and nurses, ranging from 25% to over 60% depending on the setting and specialty [18–20]. Therefore, this study was designed to evaluate the prevalence of burnout among healthcare workers in the primary healthcare clinics in Al-Khobar, Eastern Region of Saudi Arabia, and to identify associated factors.

## Materials and methods

### Study setting

This cross-sectional study was conducted between 1 January 2024 and 30 March 2024 in the primary healthcare (PHC) centers located in Al-Khobar, Eastern Province, Saudi Arabia.

### Ethical considerations

Informed consent was obtained electronically. At the beginning of the online questionnaire, participants were provided with a detailed information sheet outlining the study purpose, procedures, confidentiality measures, and their rights, including the right to withdraw at any time. Participants were required to indicate their voluntary agreement by selecting an electronic consent option before proceeding to the survey. No personal identifiers were collected, and all responses remained anonymous. Ethical approval for this study was obtained from the Institutional Review Board (IRB) of the Eastern Region – Al-Khobar (ER IRB KH), under approval number H-05-KH-103

### Study design and sampling

A non-probability convenience sampling approach was used, as all eligible staff who were available during the data collection period and agreed to participate were included in the study. The total number of PHC workers employed in the Al-Khobar cluster was not officially available; therefore, the final sample represents only those who voluntarily responded within the data collection window. The period allocated for the data collection was two months; hence, all the responses received during that period were included in the study.

### Inclusion and exclusion criteria

Inclusion criteria for the study was (1) age of a participant must be between 18 and 65 years, and (2) A participant must be healthcare staff working in the Al-Khobar health care network. The study had only one exclusion criterion, which stated that a participant who could not read and understand Arabic or English was excluded from the study.

### Data collection instruments

Data were collected through a structured electronic questionnaire consisting of three sections. The first part of the questionnaire was about the demographic characteristics of the study participants, which included age, gender, marital status, number of children, and monthly income. The second part of the questionnaire was about the professional factors, which included position, experience, work duration, patients seen per day, number of workers in the department, working hours, and working shifts.

The last part of the questionnaire consisted of the Maslach Burnout Inventory Human Services Survey (MBI-HSS). The MBI-HSS is a validated 22-item instrument with three subscales: Emotional Exhaustion (EE; 9 items), Depersonalization (DP; 5 items), and Personal Accomplishment (PA; 8 items). Responses for each item were based on a 7-point Likert scale ranging from 0 (never) to 6 (every day). The total score for each subscale was computed by summing the responses.

Subscale scores were then categorized as low, moderate, or high using the following descriptive cutoffs commonly applied in healthcare research

Emotional Exhaustion: low (≤16), moderate (17–26), high (≥27)

Depersonalization: low (≤5), moderate (6–9), high (≥10)

Personal Accomplishment: low (≤33; indicating higher burnout), moderate (34–39), high (≥40; indicating lower burnout)

These cutoffs are descriptive categories only and are not intended for clinical diagnosis. The official MBI Manual (4th edition, 2016) removed rigid diagnostic cutoffs because they lack strong validity. We used these thresholds because they are widely reported in the international healthcare literature for descriptive purposes (Brady et al., 2020; Soares et al., 2023) [21,22]. The Arabic version of the MBI-HSS used in this study has been previously validated in Arabic-speaking healthcare populations (Henchiri et al., 2025) [23].

### Data collection procedure

The questionnaire was distributed electronically via official institutional WhatsApp groups and email to all PHC staff with prior approval from the Institutional Review Board (IRB). Reminder messages were sent every 15 days to maximize response rate. While this recruitment method is standard practice within the Al-Khobar health cluster where all staff are enrolled in official institutional WhatsApp groups as part of routine operations it may not have reached providers who are less digitally active. A total of 114 valid responses were received and included in the analysis. The exact total number of eligible PHC staff in the Al-Khobar cluster was not officially available from the administration; therefore, a formal response rate could not be calculated.

### Statistical analysis

Statistical Package for the Social Sciences (SPSS v.23) was used for data entry and analysis. Frequency distribution tables were generated as part of the descriptive data analysis. Chi-square test/fisher exact test was used to study the association between the MBI subscales and the demographic and professional characteristics of the study participants. Fisher’s exact test was applied when any expected cell frequency was less than 5. Multivariable logistic regression was performed to identify independent factors associated with high burnout (defined as high Emotional Exhaustion and high Depersonalization along with low Personal Accomplishment). The model was adjusted for age, gender, monthly income, number of patients seen per day, working shifts, and years of experience. Adjusted odds ratios (OR), 95% confidence intervals (CI), and p-values are reported. All p-values less than 0.05 were considered statistically significant.

## Results

Total number of responses collected and included in the analysis were 114. The proportion of the participants who were 35 years old or more were 56.1% while 43.9% were less than 35 years old. Among the participants, 71.9% were females and 80.7% were married. Those who had up to bachelor level education were highest in proportion 48.2% followed by 27.2% who had diploma as an education. Regarding number of kids, 66.7% were having kids between 1–4 (Table 1).

**Table 1:** Demographics of the study participants.

Age	Frequency	Percent
Less than 35	50	43.9
More than equal to 35	64	56.1
**Gender**		
Male	32	28.1
Female	82	71.9
**Marital Status**		
Single	14	12.3
Married	92	80.7
Divorced	8	7.0
**Number of Kids**		
None	29	25.4
1-2	40	35.1
3-4	36	31.6
5 or more	9	7.9
**Monthly Income**		
less than 12K	22	19.3
more than 12K	92	80.7
**Education**		
Diploma	31	27.2
Bachelor	55	48.2
Master	8	7.0
PhD	20	17.5

Descriptive analysis was performed for the medical practice related questions (Table 2). Evaluating the position/designation of the participants, 36% were nurses followed by physicians (34.2%). Regarding the experience level, over half of the participants had more than 10 years of working experience. In addition, over 70% of the respondents replied that they see up to 40 patients per day (Table 2).

**Table 2:** Clinical statistics of the study participants.

Position	Frequency	Percent
Physician	39	34.2
Nurse	41	36.0
Dentist	23	20.2
technician	2	1.8
Psycologist/social work	1	0.9
Other	8	7.0
**Experience (years)**		
Less than 5	23	20.2
5-10	30	26.3
10 or more	61	53.5
**Work Duration (Years)**		
less than 1	19	16.7
1-2	23	20.2
3-5	35	30.7
More than 5	37	32.5
**Patients per day**		
Up to 20	41	36.0
20-40	40	35.1
40-60	10	8.8
More than 60	23	20.2
**Number of Worker**		
Alone	20	17.5
more than 1	94	82.5
**Working Hours**		
Full time	113	99.1
Part time	1	0.9
**Working Shifts**		
Day	71	62.3
Night	1	0.9
Both	42	36.8

Table 3 summarizes the distribution of the three Maslach Burnout Inventory subscales. Overall, 21.0% of participants reported low Emotional Exhaustion, while 79.0% had moderate or high Emotional Exhaustion. For Depersonalization, 91.2% had moderate or high levels. For Personal Accomplishment, 32.5% had low Personal Accomplishment (indicating higher burnout on this dimension).

**Table 3:** Burnout dimension statistics.

Section	Level	Frequency	Percent
**Emotional Exhaustion**	Low	24	21.0
	Moderate	45	39.5
	High	45	39.5
**Depersonalization**	Low	10	8.8
	Moderate	24	21.1
	High	80	70.2
**Personal accomplishment**	Low	37	32.5
	Moderate	25	21.9
	High	52	45.6

According to our predefined classification, 21.1% (n = 24/114) of the participants met the criteria for high burnout, defined as high Emotional Exhaustion and high Depersonalization along with low Personal Accomplishment.

Multivariable logistic regression analysis showed that female gender (OR 2.85, 95% CI 1.12–7.28, p = 0.028), monthly income less than 12,000 SAR (OR 3.41, 95% CI 1.45–8.02, p = 0.005), and seeing more than 40 patients per day (OR 2.67, 95% CI 1.08–6.60, p = 0.034) were independently associated with higher odds of burnout (Table 4).

**Table 4:** Multivariable logistic regression analysis of factors associated with high burnout (n = 114).

Variable	Category	OR	95% CI	P-value
Gender	Female vs Male	2.85	1.12–7.28	0.028*
Monthly income	<12,000 vs ≥ 12,000 SAR	3.41	1.45–8.02	0.005*
Patients seen per day	>40 vs ≤ 40	2.67	1.08–6.60	0.034*
Working shifts	Both vs Day only	2.12	0.95–4.73	0.066
Age	≥35 vs < 35 years	1.34	0.62–2.89	0.455
Years of experience	>10 vs ≤ 10	0.78	0.35–1.74	0.548

*Statistically significant at 0.05 level of significance

Prevalence of emotional exhaustion among the study participants in comparison with their demographic characteristics summarized in table 4. It was found that the female participants had significantly high level of exhaustion (46.3%) compared to males (21.9%) with the p-value 0.043. It was also found the monthly income was causing the emotional exhaustion as well. Those who had monthly income less than 12K had significantly high (68.2%) level of exhaustion compared to those with more than 12K monthly income (32.6%) (p = 0.009). When level of education was compared with the level of exhaustion it was found that those who had education up to diploma had significantly highly (64.5%) emotionally exhausted among the others with education level more than diploma (p = 0.024). Study of the relationship between number of patients seen per day with the emotional exhaustion level provided that those seeing more than 60 patients per day had significantly highly exhausted compared to those seeing lesser number of patients (p = 0.032). Emotional exhaustion was also found high among those having duty in shifts. Those who were working in the day shifts only had 31% of high level of exhaustion however those who were working in both sifts had 54.8% and the difference in the proportion was significantly different (p = 0.043) (Table 5).

**Table 5:** Association of level of Emotional exhaustion with the demographic variables.

Gender	Emotional Exhaustion			P-value
Low	Moderate	High
Male	10(31.3)	15(46.9)	7(21.9)	0.043*
Female	14(17.1)	30(36.6)	38(46.3)	
**Monthly Income**				
Less than 12K	3(13.6)	4(18.2)	15(68.2)	0.009*
More than 12K	21(22.8)	41(44.6)	30(32.6)	
**Education level**				
Diploma	4(12.9)	7(22.6)	20(64.5)	0.024*
Bachelor	11(20.0)	26(47.3)	18(32.7)	
Master	4(50.0)	3(37.5)	1(12.5)	
PhD	5(25.0)	9(45.0)	6(30.0)	
**Experience (Yrs)**				
Less than 5	4(17.4)	10(43.5)	9(39.1)	0.710
5-10	7(23.3)	14(46.7)	9(30.0)	
More than 10	13(21.3)	21(34.4)	27(44.3)	
**Patients per Day**				
Up to 20	14(34.1)	16(39.0)	11(26.8)	0.032*
20-40	6(15.0)	20(50.0)	14(35.0)	
40-60	2(20.0)	3(30.0)	5(50.0)	
More than 60	2(8.7)	6(26.1)	15(65.2)	
**Working Shifts**				
Day	20(28.2)	29(40.8)	22(31.0)	0.043*
Both	4(9.5)	15(35.7)	23(54.8)	

*Statistically significant at 0.05 level of significance

Association of the level of depersonalization was studied with the demographic variables and it was found that the gender and work duration had significant association with depersonalization (Table 5). It was found that the 78% of the females had high level of depersonalization and 50% of males had high level of depersonalization which was significantly different (p = 0.011). Similarly, when working duration was studied with depersonalization level, it was found that 80% of participants with 3–5 years of working had high level of depersonalization compared to the other levels of work duration (p = 0.007) (Table 6).

**Table 6:** Association of level of depersonalization with the demographic variables.

Gender	Depersonalization			P-value
Low	Moderate	High	
Male	4(12.5)	12(37.5)	16(50.0)	0.011*
Female	6(7.3)	12(14.6)	64(78.0)	
**Experience (Yrs)**				
Less than 5	2(8.7)	6(26.1)	15(65.2)	0.919
5-10	3(10.0)	7(23.3)	20(66.7)	
More than 10	5(8.2)	11(18.0)	45(73.8)	
**Work duration (yrs)**				
Less than 1	5(26.3)	1(5.3)	13(68.4)	0.007*
1-2	0	9(39.1)	14(60.9)	
3-5	1(2.9)	6(17.1)	28(80.0)	
More than 5	4(10.8)	8(21.6)	25(67.6)	

*Statistically significant at 0.05 level of significance

Similarly, personal accomplishment was analysed with the demographic characteristics and found that the number of kids caused significant change in the level of personal accomplishment. It was observed that the increase in the number of kids causing decrease in personal accomplishment level. On the contrary, participants with 1–2 kids had significantly high level of personal accomplishment compared to those with more or no kids (p = 0.001) (Table 7).

**Table 7:** Association of level of personal accomplishment with the demographic variables.

Gender	Personal accomplishment			P-value
High	Moderate	Low
Male	19(59.4)	5(15.6)	8(25.0)	0.181
Female	33(40.2)	20(24.4)	29(35.4)	
**Number of kids**				
None	15(51.7)	7(24.1)	7(24.1)	0.001*
1-2	25(62.5)	5(12.5)	10(25.0)	
3-4	12(33.3)	12(33.3)	12(33.3)	
5 or more	0	1(11.1)	8(88.9)	
**Experience (Yrs)**				
Less than 5	13(56.5)	5(21.7)	5(21.7)	0.606
5-10	15(50.0)	6(20.0)	9(30.0)	
More than 10	24(39.3)	14(23.0)	23(37.7)	

*Statistically significant at 0.05 level of significance

## Discussion

Burnout among healthcare workers has increasingly become a global public health concern, particularly in primary care settings where workload, emotional demands, and patient interaction are high. Although technological and clinical advances have transformed modern healthcare, the psychological burden on frontline workers continues to rise. Therefore, evaluation of the mental health and burnout syndrome in the healthcare providers has now become an area of interest among the researchers [24–26]. The present study contributes to this gap by assessing the prevalence and associated factors of burnout among primary healthcare workers in Al-Khobar, Eastern Region of Saudi Arabia.

The findings showed that 21.1% of the participants met the criteria for high burnout (defined as high Emotional Exhaustion and high Depersonalization along with low Personal Accomplishment). In terms of individual dimensions, 70.2% reported high depersonalization, 39.5% reported high emotional exhaustion, and 32.5% had low Personal Accomplishment. These results are comparable to an Iranian study that reported a 17.3% burnout prevalence among PHC staff, but higher than findings from Ecuador (2.6%) and Brazil (7%) [27–29]. Conversely, other studies have documented substantially higher rates, such as 52.9% burnout in Iranian PHC workers [30] and 32% among family medicine residents in Al-Madina, Saudi Arabia [15]. Such variability may reflect differences in workload intensity, staffing levels, organisational support, cultural coping styles, and burnout measurement criteria across countries [19,20].

With regard to burnout dimensions, the prevalence of high depersonalization (70.2%) in this study was higher than that reported in Iran (49.6%), Palestine (38%), and Malawi (22.8%) [27,31,32]. The prevalence of high emotional exhaustion (39.5%) was consistent with many previously published studies. In the Iranian PHC study, the prevalence of emotional exhaustion was 35.7%, and among Malawian health workers it was 32.7% [27,32]. A systematic review of medical residents also reported a similar emotional exhaustion rate of 38.9% [33].

Study of the dimensions of the burnout with the demographic variables provided that the prevalence of burnout was significantly high among the females compared to males. The similar findings were reported in the studies published by Amiri et al and Zarei et al, in which they found significantly high prevalence of burnout among females compared to males [27,30]. However, the difference in the proportions was not as high as it was found in the present study. In addition, many other studies also reported the high prevalence of emotional exhaustion among the female healthcare workers compared to males [15,30,34]. Analysis of the data was provided that the monthly was another factor causing the emotional exhaustion among the study participants. It was found that the less monthly income increases emotional exhaustion significantly. These findings were consistent with previously published studies in which less income or dissatisfaction with the income causing significant increase in the burnout [27,34].

Many studies found the years of working experience had a significant association with the burnout syndrome among healthcare providers. Studies found that burnout was more prevalent among those with more working experience compared to their counterparts [35,36]. Contrarily, some studies found that the burnout was highly prevalent among those with less working experience [29,37,38]. However, in the present study, a statistically insignificant relationship between the years of working experience and prevalence of burnout syndrome was found.

## Limitations

The present study had some limitations which prevented us from generalizing the study findings. First, its cross-sectional design prevents causal inference between contributing factors and burnout outcomes. Second, we used a non-probability convenience sampling method and distributed the questionnaire via WhatsApp and official email, which may have introduced selection bias and volunteer/response bias; participants who agreed to respond might differ from non-respondents. Additionally, the use of electronic distribution channels may have introduced digital access bias, potentially under-representing providers who are less digitally active or have limited engagement with institutional communication platforms. Third, the study relied on self-reported data using the Maslach Burnout Inventory, making the results susceptible to recall bias and social desirability bias. Specifically, healthcare providers may under-report burnout symptoms due to professional stigma or fear of judgment, potentially leading to an underestimation of true prevalence. Future studies incorporating objective markers of occupational stress alongside self-reported tools would provide a more comprehensive picture. Fourth, the sample size was relatively small (n = 114), which may limit the statistical power of subgroup analyses and the precision of effect estimates in the multivariable logistic regression. Results particularly odds ratios with wide confidence intervals should be interpreted cautiously. Future studies with larger, multi-site, or population-based samples are needed to confirm these associations and enhance generalizability across the broader Saudi primary healthcare workforce. Possible measures include workload redistribution, psychological support programmers, regular screening using validated tools such as the MBI, and financial or role-based incentives for high-demand positions. Furthermore, the exact number of eligible PHC staff in the Al-Khobar cluster was not officially available; therefore, a formal response rate could not be calculated, which precludes a definitive assessment of non-response bias.

## Conclusion

The prevalence of burnout among the primary healthcare workers was found to be 21.1% which was comparatively less compared to most of the studies. However, there were few factors which were causing the burnout among the study participants. Some of those factors were the gender, monthly income and per day patients load. Therefore, it is required to take necessary measures that would help to reduce the prevalence of burnout syndrome especially focusing on the factors that were causing the burnout.
